# Supplementary material for: Early integration of pastoralism and millet cultivation in Bronze Age Eurasia
Source: Proc Biol Sci. 2019 Sep 4;286(1910):20191273. doi: 10.1098/rspb.2019.1273 (PMC6743000; doi:10.1098/rspb.2019.1273)
Supplement: Supplementary Information [file rspb20191273supp1.docx]

**Early integration of pastoralism and millet cultivation in Bronze Age Eurasia**

*Proceedings of the Royal Society B*

DOI: 10.1098/rspb.2019.1273

**Supplementary Information**

Taylor R. Hermes^1,2^*, Michael D. Frachetti^3^*, Paula N. Doumani Dupuy^2,4^, Alexei Mar’yashev^5^†, Almut Nebel^1,6^, Cheryl A. Makarewicz^1,2^*

^1^Graduate School “Human Development in Landscapes”, Kiel University, Leibniz Straße 3, 24118, Kiel, Germany

^2^Institute of Prehistoric and Protohistoric Archaeology, Kiel University, Johanna-Mestorf-Straße 2-6, 24118, Kiel, Germany

^3^Department of Anthropology, Washington University in St. Louis, One Brookings Drive, St. Louis, 63130, USA

^4^School of Humanities and Social Sciences, Nazarbayev University, Kabanbay Batyr Avenue 53, Astana, 010000, Kazakhstan

^5^Margulan Institute of Archaeology, Dostyk Avenue 44, Almaty, 480100, Kazakhstan

^6^Institute of Clinical Molecular Biology, Kiel University, University Hospital Schleswig-Holstein, Rosalind-Franklin Straße 12, 24105, Kiel, Germany

*Corresponding authors:

Taylor Hermes, [trhermes@gshdl.uni-kiel.de](mailto:trhermes@gshdl.uni-kiel.de)

Michael Frachetti, [frachetti@wustl.edu](mailto:frachetti@wustl.edu)

Cheryl Makarewicz, [c.makarewicz@ufg.uni-kiel.de](mailto:c.makarewicz@ufg.uni-kiel.de)

†Deceased 12 March 2018

**Note:** References in this Supplementary Information are independent from references in the main text. References cited in datasets refer those in the Supplementary Information.

**Supplemental Text 1.** PCR amplifications were performed in a final volume of 25 μL, containing 1X Immolase buffer, 1.5 mM MgCl_2_, 4% DMSO, 200 µM each dNTP, 0.4 µM each primer, 1 U Immolase DNA Polymerase, and 2 μL aDNA extract as template. Reactions were performed in duplicates on each extract and included PCR blanks for every five samples, undergoing initialization at 94°C for 10 min, 42 cycles of 94°C for 30 s, 50°C for 30 s, 72°C for 30 s, and a final elongation step at 72°C for 10 min. Quality of PCR products was checked on a QIAxcel system, and products with single bands of expected size, with uncontaminated accompanying negative controls, were sequencing with the BigDye Terminator v3.1 Kit (Thermo Fisher Scientific), following the manufacturer’s instructions. Sequencing products were purified with the DyeEx 2.0 Spin Kit (Qiagen) and analysed using the ABI Prism 310 and 3130 Genetic Analysers (Thermo Fisher Scientific). Sample and reference sequences were manually aligned (dataset S1), and a minimum spanning haplotype network was generated using POPART v. 1.7. [1] and is displayed below in figure S1.

**Supplemental Text 2.** C_4_ plant dietary sources were represented by δ^13^C values measured on modern leaves of broomcorn millet plants across China (n=17) [2]. δ^13^C values from modern samples were corrected for the Suess effect with an offset of +1.5‰ [3]. Bioapatite δ^13^C values were converted to dietary δ^13^C values using a trophic enrichment factor (TEF), also known as diet-tissue spacing factor, for ruminants of 14‰ [4,5], including δ^13^C values of saiga summer hair [6]. C_3_ sources were modelled using a TEF of 14‰ for bioapatite [4,5], and for broomcorn millet a TEF of 12‰ was used for caprines [7]. For cattle and red deer bioapatite, 14‰ was used for both sources [4,5]. The TEF used for bone collagen of all ruminant taxa was 5‰ [8,9]. The TEF used for swine bone collagen was 4‰ [10], and for steppe tortoise bone collagen 1‰ was used [11]. MixSIAR models were run with on three chains using uniform priors at 300,000 iterations with a burn-in of 200,000, followed by a 1:100 thinning.

**Table S1.** Radiocarbon dates for Dali, Tasbas, and Begash modelled according to archaeological strata. Radiocarbon samples Beta-484122* (Dali) and Poz-88835* (Begash), respectively correspond to aDNA samples KO180909 and KO180907, and also isotope tooth specimens T-027 and T-032.

| **Site** |  | **Sample ID** | **Material** | **Raw date (BP)** | **±** |  | **Unmodelled (BC/AD)** | | |  | **Modelled (BC/AD)** | | | **Reference** |
| --- | --- | --- | --- | --- | --- | --- | --- | --- | --- | --- | --- | --- | --- | --- |
| Phase |  |  |  |  |  |  |  |  |  |  |  |  |  |  |
| Context |  |  |  |  |  |  | from | to | mean |  | from | to | mean |  |
|  |  |  |  |  |  |  |  |  |  |  |  |  |  |  |
| **Dali** |  |  |  |  |  |  |  |  |  |  |  |  |  |  |
|  |  |  |  |  |  |  |  |  |  |  |  |  |  |  |
| Start Phase 1 |  |  |  |  |  |  |  |  |  |  | -2855 | -2600 | -2705 |  |
| Pithouse |  |  |  |  |  |  |  |  |  |  |  |  |  |  |
| Walls |  |  |  |  |  |  |  |  |  |  |  |  |  |  |
|  |  | OS-99768 | charcoal | 4180 | 25 |  | -2885 | -2670 | -2775 |  | -2790 | -2600 | -2680 | This study |
|  |  | OS-99766 | charcoal | 4090 | 25 |  | -2860 | -2500 | -2670 |  | -2830 | -2590 | -2655 | This study |
| Floors |  |  |  |  |  |  |  |  |  |  |  |  |  |  |
|  |  | PSUAMS-2071 | human bone collagen | 4075 | 25 |  | -2850 | -2490 | -2635 |  | -2665 | -2580 | -2620 | [12] |
|  |  | **Beta-484122*** | sheep dental collagen | 4080 | 30 |  | -2855 | -2495 | -2645 |  | -2665 | -2580 | -2620 | This study |
|  |  | ISGS-A2837 | charcoal | 4100 | 20 |  | -2855 | -2575 | -2685 |  | -2635 | -2570 | -2600 | This study |
|  |  | ISGS-A2829 | charcoal | 4045 | 20 |  | -2625 | -2485 | -2555 |  | -2625 | -2495 | -2570 | This study |
| Terrace |  |  |  |  |  |  |  |  |  |  |  |  |  |  |
|  |  | Poz-92105 | cervid bone collagen | 4040 | 35 |  | -2835 | -2470 | -2570 |  | -2665 | -2500 | -2590 | This study |
| End Phase 1 |  |  |  |  |  |  |  |  |  |  | -2625 | -2440 | -2545 |  |
|  |  |  |  |  |  |  |  |  |  |  |  |  |  |  |
| Start Break |  |  |  |  |  |  |  |  |  |  | -2560 | -2210 | -2370 |  |
|  |  | OS-99857 | charcoal | 3820 | 30 |  | -2450 | -2140 | -2265 |  | -2405 | -2140 | -2260 | This study |
| End Break |  |  |  |  |  |  |  |  |  |  | -2330 | -1660 | -2060 |  |
|  |  |  |  |  |  |  |  |  |  |  |  |  |  |  |
| Start Phase 2 |  |  |  |  |  |  |  |  |  |  | -1760 | -1545 | -1645 |  |
|  |  | ISGS-A2838 | charcoal | 3330 | 15 |  | -1665 | -1530 | -1615 |  | -1650 | -1530 | -1600 | This study |
|  |  | Poz-92107 | cattle bone collagen | 3330 | 30 |  | -1690 | -1525 | -1610 |  | -1660 | -1525 | -1595 | This study |
|  |  | OS-93009 | charcoal | 3320 | 30 |  | -1685 | -1520 | -1595 |  | -1655 | -1525 | -1590 | This study |
|  |  | Poz-92106 | horse bone collagen | 3240 | 30 |  | -1615 | -1435 | -1515 |  | -1620 | -1465 | -1560 | This study |
|  |  | Poz-92109 | caprine bone collagen | 3240 | 30 |  | -1615 | -1435 | -1515 |  | -1620 | -1465 | -1560 | This study |
| End Phase 2 |  |  |  |  |  |  |  |  |  |  | -1615 | -1410 | -1520 |  |
|  |  |  |  |  |  |  |  |  |  |  |  |  |  |  |
|  |  |  |  |  |  |  |  |  |  |  |  |  |  |  |
| **Tasbas** |  |  |  |  |  |  |  |  |  |  |  |  |  |  |
|  |  |  |  |  |  |  |  |  |  |  |  |  |  |  |
| Start Phase 1 |  |  |  |  |  |  |  |  |  |  | -2855 | -2500 | -2655 |  |
|  |  | OS-93050 | charcoal | 4100 | 30 |  | -2865 | -2500 | -2690 |  | -2700 | -2490 | -2600 | [13] |
|  |  | OS-93054 | charcoal | 4060 | 30 |  | -2840 | -2480 | -2605 |  | -2660 | -2490 | -2575 | [13] |
|  |  | Beta-391200 | wheat | 4010 | 30 |  | -2620 | -2465 | -2530 |  | -2620 | -2475 | -2545 | [14] |
| End Phase 1 |  |  |  |  |  |  |  |  |  |  | -2620 | -2290 | -2480 |  |
|  |  |  |  |  |  |  |  |  |  |  |  |  |  |  |
| Start Break |  |  |  |  |  |  |  |  |  |  | -2495 | -1975 | -2210 |  |
|  |  | OS-93268 | charcoal | 3670 | 45 |  | -2200 | -1925 | -2055 |  | -2200 | -1925 | -2055 | [14] |
| End Break |  |  |  |  |  |  |  |  |  |  | -2135 | -1470 | -1850 |  |
|  |  |  |  |  |  |  |  |  |  |  |  |  |  |  |
| Start Phase 2a |  |  |  |  |  |  |  |  |  |  | -1705 | -1300 | -1465 |  |
|  |  | OS-93053 | charcoal | 3150 | 35 |  | -1505 | -1305 | -1420 |  | -1495 | -1300 | -1390 | [13] |
|  |  | OS-92277 | barley | 3090 | 40 |  | -1440 | -1230 | -1345 |  | -1435 | -1275 | -1355 | [13] |
|  |  | OS-91990 | barley | 3030 | 35 |  | -1405 | -1130 | -1290 |  | -1410 | -1240 | -1335 | [13] |
| End Phase 2a |  |  |  |  |  |  |  |  |  |  | -1395 | -1170 | -1285 |  |
|  |  |  |  |  |  |  |  |  |  |  |  |  |  |  |
| Start Phase 2b |  |  |  |  |  |  |  |  |  |  | -1285 | -1070 | -1180 |  |
|  |  | OS-97173 | charcoal | 2940 | 30 |  | -1260 | -1040 | -1145 |  | -1210 | -1055 | -1135 | [14] |
|  |  | OS-93052 | charcoal | 2930 | 30 |  | -1220 | -1025 | -1130 |  | -1210 | -1050 | -1135 | [14] |
|  |  | OS-93051 | charcoal | 2920 | 25 |  | -1210 | -1025 | -1115 |  | -1210 | -1050 | -1130 | [14] |
| End Phase 2b |  |  |  |  |  |  |  |  |  |  | -1195 | -960 | -1080 |  |
|  |  |  |  |  |  |  |  |  |  |  |  |  |  |  |
| Start Phase 3 |  |  |  |  |  |  |  |  |  |  | -1090 | -825 | -945 |  |
|  |  | OS-97172 | charcoal | 2720 | 35 |  | -930 | -805 | -870 |  | -970 | -805 | -875 | [14] |
| End Phase 3 |  |  |  |  |  |  |  |  |  |  | -970 | -530 | -790 |  |
|  |  |  |  |  |  |  |  |  |  |  |  |  |  |  |
|  |  |  |  |  |  |  |  |  |  |  |  |  |  |  |
| **Begash** |  |  |  |  |  |  |  |  |  |  |  |  |  |  |
|  |  |  |  |  |  |  |  |  |  |  |  |  |  |  |
| Start Phase 1a |  |  |  |  |  |  |  |  |  |  | -2525 | -2210 | -2345 |  |
|  |  | AA52926 | charcoal | 4220 | 220 |  | -3500 | -2205 | -2835 |  | -2480 | -2200 | -2305 | [15] |
|  |  | OS-54364 | charcoal | 3870 | 35 |  | -2470 | -2205 | -2355 |  | -2390 | -2150 | -2260 | [15] |
|  |  | Beta-266458 | wheat + broomcorn millet | 3840 | 40 |  | -2465 | -2150 | -2310 |  | -2400 | -2140 | -2260 | [16] |
|  |  | **Poz-88835*** | sheep bone collagen | 3830 | 30 |  | -2460 | -2150 | -2285 |  | -2350 | -2145 | -2255 | This study |
|  |  | OS-54365 | charcoal | 3800 | 35 |  | -2430 | -2060 | -2240 |  | -2335 | -2135 | -2230 | [15] |
|  |  | Beta-266459 | charcoal | 3760 | 40 |  | -2295 | -2035 | -2175 |  | -2295 | -2050 | -2190 | [16] |
|  |  | Beta-266460 | charcoal | 3740 | 40 |  | -2285 | -2025 | -2140 |  | -2285 | -2045 | -2170 | [16] |
|  |  | Beta-266457 | charcoal | 3720 | 40 |  | -2275 | -1980 | -2115 |  | -2280 | -2035 | -2155 | [16] |
|  |  | OS-54334 | charcoal | 3650 | 45 |  | -2145 | -1895 | -2030 |  | -2275 | -1990 | -2125 | [15] |
| End Phase 1a |  |  |  |  |  |  |  |  |  |  | -2200 | -1925 | -2080 |  |
|  |  |  |  |  |  |  |  |  |  |  |  |  |  |  |
| Start Phase 1b |  |  |  |  |  |  |  |  |  |  | -2005 | -1755 | -1875 |  |
|  |  | OS-54833 | charcoal | 3540 | 140 |  | -2290 | -1525 | -1900 |  | -1940 | -1715 | -1825 | [15] |
|  |  | OS-54328 | charcoal | 3500 | 30 |  | -1910 | -1740 | -1820 |  | -1890 | -1750 | -1820 | [15] |
|  |  | OS-54333 | charcoal | 3490 | 30 |  | -1895 | -1695 | -1815 |  | -1885 | -1750 | -1820 | [15] |
|  |  | OS-54329 | charcoal | 3460 | 35 |  | -1885 | -1690 | -1785 |  | -1885 | -1735 | -1815 | [15] |
| End Phase 1b |  |  |  |  |  |  |  |  |  |  | -1875 | -1660 | -1770 |  |
|  |  |  |  |  |  |  |  |  |  |  |  |  |  |  |
| Start Phase 2 |  |  |  |  |  |  |  |  |  |  | -1795 | -1525 | -1655 |  |
|  |  | OS-54367 | charcoal | 3310 | 35 |  | -1685 | -1505 | -1585 |  | -1660 | -1500 | -1570 | [15] |
|  |  | OS-54000 | charcoal | 3100 | 50 |  | -1495 | -1225 | -1355 |  | -1495 | -1225 | -1355 | [15] |
|  |  | OS-54369 | charcoal | 2880 | 40 |  | -1210 | -930 | -1060 |  | -1220 | -970 | -1095 | [15] |
| End Phase 2 |  |  |  |  |  |  |  |  |  |  | -1195 | -730 | -975 |  |
|  |  |  |  |  |  |  |  |  |  |  |  |  |  |  |
| Start Phase 3a |  |  |  |  |  |  |  |  |  |  | -1030 | -515 | -775 |  |
|  |  | AA52925 | charcoal | 2657 | 84 |  | -1020 | -540 | -820 |  | -910 | -470 | -690 | [15] |
|  |  | AA52927 | charcoal | 2435 | 47 |  | -760 | -400 | -570 |  | -765 | -425 | -605 | [15] |
|  |  | OS-54366 | charcoal | 2430 | 45 |  | -755 | -400 | -565 |  | -760 | -420 | -605 | [15] |
| End Phase 3a |  |  |  |  |  |  |  |  |  |  | -725 | -275 | -490 |  |
|  |  |  |  |  |  |  |  |  |  |  |  |  |  |  |
| Start Phase 3b |  |  |  |  |  |  |  |  |  |  | -390 | -175 | -260 |  |
|  |  | AA52920 | charcoal | 2253 | 35 |  | -400 | -205 | -300 |  | -310 | -165 | -230 | [15] |
|  |  | AA52923 | charcoal | 2152 | 36 |  | -360 | -55 | -220 |  | -345 | -85 | -180 | [15] |
|  |  | OS-54376 | charcoal | 2150 | 30 |  | -360 | -60 | -220 |  | -345 | -90 | -180 | [15] |
|  |  | AA52924 | charcoal | 2142 | 41 |  | -360 | -50 | -200 |  | -340 | -55 | -170 | [15] |
|  |  | AA52922 | charcoal | 2141 | 35 |  | -355 | -50 | -195 |  | -340 | -55 | -170 | [15] |
|  |  | OS-54368 | charcoal | 2140 | 30 |  | -355 | -55 | -195 |  | -340 | -60 | -170 | [15] |
|  |  | OS-54492 | charcoal | 2090 | 35 |  | -205 | -1 | -115 |  | -210 | -90 | -160 | [15] |
|  |  | OS-54377 | charcoal | 2060 | 35 |  | -175 | 20 | -80 |  | -195 | -55 | -130 | [15] |
|  |  | OS-54818 | charcoal | 2050 | 80 |  | -355 | 125 | -80 |  | -190 | -15 | -105 | [15] |
| End Phase 3b |  |  |  |  |  |  |  |  |  |  | -185 | 30 | -80 |  |
|  |  |  |  |  |  |  |  |  |  |  |  |  |  |  |
| Start Phase 4 |  |  |  |  |  |  |  |  |  |  | -75 | 185 | 65 |  |
|  |  | AA52918 | charcoal | 1874 | 37 |  | 60 | 235 | 140 |  | 70 | 235 | 155 | [15] |
|  |  | AA52917 | charcoal | 1853 | 40 |  | 65 | 250 | 165 |  | 75 | 255 | 175 | [15] |
|  |  | AA52916 | charcoal | 1852 | 53 |  | 30 | 325 | 165 |  | 65 | 325 | 180 | [15] |
|  |  | OS-54331 | charcoal | 1830 | 40 |  | 75 | 325 | 185 |  | 85 | 325 | 190 | [15] |
|  |  | OS-54363 | charcoal | 1700 | 35 |  | 250 | 410 | 335 |  | 250 | 410 | 335 | [15] |
|  |  | OS-54330 | charcoal | 1600 | 35 |  | 390 | 545 | 470 |  | 345 | 540 | 440 | [15] |
| End Phase 4 |  |  |  |  |  |  |  |  |  |  | 350 | 775 | 525 |  |
|  |  |  |  |  |  |  |  |  |  |  |  |  |  |  |
| Start Phase 5 |  |  |  |  |  |  |  |  |  |  | 680 | 1380 | 1120 |  |
|  |  | AA52921 | charcoal | 715 | 33 |  | 1225 | 1385 | 1290 |  | 1245 | 1390 | 1295 | [15] |
|  |  | OS-54378 | charcoal | 575 | 30 |  | 1300 | 1420 | 1360 |  | 1300 | 1420 | 1350 | [15] |
| End Phase 5 |  |  |  |  |  |  |  |  |  |  | 1305 | 1705 | 1470 |  |
|  |  |  |  |  |  |  |  |  |  |  |  |  |  |  |
| Start Phase 6 |  |  |  |  |  |  |  |  |  |  | 1550 | 1920 | 1730 |  |
|  |  | OS-54160 | charcoal | 135 | 35 |  | 1665 | 1945 | 1805 |  | 1670 | 1935 | 1795 | [15] |
|  |  | OS-54383 | charcoal | 110 | 25 |  | 1680 | 1935 | 1820 |  | 1680 | 1930 | 1795 | [15] |
|  |  | OS-54375 | charcoal | 100 | 30 |  | 1680 | 1935 | 1820 |  | 1680 | 1930 | 1795 | [15] |
| End Phase 6 |  |  |  |  |  |  |  |  |  |  | 1685 | 2030 | 1855 |  |

**Table S2.** Ancient DNA sample list. Successful recovery of mitochondrial cytochrome *b* sequences for each sample is indicated by species and genus (e.g., *Capra hircus*) in the column for Genetic taxon. DNA sequences for study samples and reference taxa used to generate Fig. 2 are in FASTA format in dataset S1.

| **Sample ID** | **Site** | **Genetic taxon** | **Zooarch taxon** | **Taxon conflict** | **Sample type** | **Specimen ID / context info** |
| --- | --- | --- | --- | --- | --- | --- |
| KG160125 | Begash | *Capra hircus* | *Capra hircus* |  | tooth; dp/4 | T-068; IVB;g112;ctx5;phase3b |
| KG160126 | Dali | *Capra hircus* | *Capra hircus* |  | tooth; P/4 | T-051; OP1;d1;ctx5; phase 2 |
| KG160127 | Tasbas | *Capra hircus* | *Capra hircus* |  | tooth; P/4 | T-066; 14a;ctx10; phase 2b |
| KG160128 | Tasbas | *Ovis aries* | dom. caprine |  | tooth; P/3 | T-039; 16e;ctx106; phase 2a |
| KG160129 | Tasbas | *Capra hircus* | *Capra hircus* |  | tooth; P/4 | T-055; 13a;ctx17; phase 2b |
| KG160130 | Begash | *Capra hircus* | *Capra hircus* |  | tooth; P/4 | T-067; IVB;g112;ctx5;phase3b |
| KG160131 | Dali | *Capra hircus* | *Capra hircus* |  | tooth; dp/4 | T-60; OP2;j4;ctx21; phase 1 |
| KG160132 | Dali | *Capra hircus* | *Capra hircus* |  | bone; horn core | T-47; OP3;h99;ctx19; phase 2 |
| KG160133 | Tasbas | *Capra hircus* | *Capra hircus* |  | bone; horn core | T-46; 15/16e;cut into 102; phase 2b |
| KG160134 | Dali | *Capra hircus* | *Capra hircus* |  | tooth; dp/4 | T-61; OP3;h99;ctx19; phase 2 |
| KG160135 | Tasbas | *Capra hircus* | *Capra hircus* |  | tooth; P/3 | T-65; 13d; under rocks; phase 2b |
| KG160136 | Begash | *Capra hircus* | *Capra hircus* |  | tooth; dp/4 | T-62; VC;e118;ctx9; phase 2 |
| KG160137 | Dali | *Capra sibirica* | *Capra sibirica* |  | petrous bone | T-69; OP3;i100;ctx19; phase 2 |
| KG170112 | Begash | *Ovis aries* | *Capra hircus* | Y | bone; distal tibia | BG0084; ASIL: 5289; VC; d116; ctx 14; phase 1b |
| KG170113 | Dali | *Ovis aries* | dom. caprine? |  | tooth; P/4; P/3 | T-53; DL-OP2; I1; ctx 24; phase 1 |
| KG170117 | Begash | *Capra hircus* | *Capra hircus* |  | bone; PH1 | BG0185; ASIL: 5631; IID; c112; ctx 11; phase 2 |
| KG170123 | Begash | Fail | *Capra hircus* |  | bone; horn core | BG0113; ASIL: 5274; VC; e118; ctx 8; phase 1b |
| KG170129 | Begash | *Capra hircus* | *Capra hircus* |  | bone; ulna | BG0100; ASIL: 5300; IB; y101; ctx 5; phase 1b |
| KG170130 | Begash | *Capra hircus* | *Capra hircus* |  | bone; ph1 | BG0145; ASIL: 5658; ID; c101; ctx 6; phase 2 |
| KG170132 | Begash | Fail | dom. caprine? |  | tooth; right M/3 | T-001; IID; a115; ctx 9; phase 1b |
| KG170133 | Begash | Fail | dom. caprine? |  | tooth; right P/3 | T-002b; IID; b115; ctx 9; phase 1b |
| KG170134 | Begash | Fail | *Capra sibirica* |  | bone (burn); tibia | BG0026; IC; b100; ctx 14; phase 1a |
| KG170135 | Begash | Fail | *Capra sibirica* |  | bone; ph1 | BG0200; IA; y099; ctx 3; phase 3a |
| KG170136 | Begash | *Capra hircus* | *Capra hircus* |  | bone; humerus | BG0294; ASIL: 5785; IID; b111; ctx 7; phase 3b |
| KG170137 | Begash | *Ovis aries* | *Capra sibirica* | Y | bone; astragalus | BG0376; IIIB; f103; ctx 3; phase 5 |
| KG170138 | Begash | Fail | *Capra hircus* |  | bone; astragalus | BG0141;ID; a104; ctx 6; phase 2 |
| KG170139 | Begash | *Ovis aries* | *Capra sibirica* | Y | bone; cervical vert | BG0206; ASIL: 5676; IA; y100; ctx 3; phase 3a |
| KG170140 | Begash | Fail | *Capra sibirica* |  | bone; calcaneus | BG0442; VC; e119; ctx 2; phase 6 |
| KO180905 | Dali | *Ovis aries* | *Ovis aries?* |  | tooth (dp/4, left) | T-59; DL-OP2; J4; ctx 21; phase 1 |
| KO180906 | Dali | *Ovis aries* | *Ovis aries?* |  | tooth (M/1, left) | T-53c; DL-OP2; I1; ctx 24; phase 1 |
| KO180907 | Begash | *Ovis aries* | *Ovis aries?* |  | tooth (P/4, right) | BG0023; IC; A100; ctx 14; phase 1a |
| KO180908 | Begash | *Ovis aries* | *Ovis aries?* |  | Tooth; M/3 | T-031; VC; C120; ctx 17; phase 1a |
| KO180909 | Dali | *Ovis aries* | *Ovis aries?* |  | tooth (M/3, left) | T-027; OP2; J6; ctx 29; phase 1 |
| KO180910 | Dali | *Ovis aries* | *Ovis aries?* |  | tooth (M/3, left) | T-050; OP3; H99; ctx 19; phase 1 |
| KO180911 | Dali | *Capra hircus* | dom. caprine? |  | tooth (M/3, left) | T-022; OP2; J5; ctx 20; phase 1 |
| KO180912 | Dali | *Ovis aries* | dom. caprine? |  | tooth (M/2, right) | T-085; OP2; I1; ctx 21; phase 1 |
| KO180913 | Tasbas | *Ovis aries* | *Ovis aries* |  | tooth (M/3, right) | T-025; 13C; ctx 20; phase 2b |
| KO180914 | Begash | Fail | dom. caprine |  | tooth (M/3, right) | T-008; VI-A; F116; ctx 11; phase 1b |
| KO180915 | Dali | *Ovis aries* | *Ovis aries* |  | tooth (M/3, left) | T-026; OP2; I6; ctx 17; phase 2 |
| KO180916 | Begash | *Ovis aries* | *Ovis aries* |  | tooth (M/3, right) | T-030; IID; A112; ctx 7; phase 3b |
| KO180919 | Begash | Fail | dom. caprine |  | tooth (M/3, right) | T112; IID; a115; ctx 11; phase 2 |
| KO181197 | Begash | *Ovis aries* | dom. caprine |  | tooth (M/3) | T-005; IID; D111; ctx 9; phase 1b |
| KO181198 | Begash | Fail | dom. caprine |  | tooth (M/2) | T-009; IA; Y099; ctx 10; phase 1b |
| KO181199 | Begash | Fail | dom. caprine |  | tooth (M/3, left) | T-002; IID; B115; ctx 9; phase 1b |
| KO181200 | Begash | *Ovis aries* | *Ovis aries* |  | tooth (dp3, left) | T-006; VC; C117; ctx 11; phase 1b |
| KO181201 | Begash | *Ovis aries* | dom. caprine |  | tooth (M2) | T-004; VC; C119; ctx 16; phase 1b |
| KO181202 | Begash | Fail | dom. caprine |  | tooth (M/2, left) | T-015; VIA; F117; ctx 4; phase 3b |
| KO181203 | Begash | *Ovis aries* | dom. caprine |  | tooth (incisor) | T-001; IID; A115; ctx 9; phase 1b |
| KO181204 | Begash | Fail | dom. caprine |  | tooth (molar) | T-001; IID; A115; ctx 9; phase 1b |
| KO181205 | Begash | *Ovis aries* | dom. caprine |  | tooth (M1, left) | T-007; ID; B101; ctx 8; phase 1b |
| KO181206 | Begash | Fail | dom. caprine |  | tooth (molar) | T-017; VIA; G117; ctx 8; phase 3b |
| KO181207 | Begash | Fail | dom. caprine |  | tooth (M/1, left) | T-107; IID; C113; ctx 15; phase 1b |
| KO181295 | Begash | Fail | *Ovis aries* |  | tooth (dp3, left) | T-070; VC; d116; ctx 14; phase 1b |
| KO181937 | Dali | *Capra hircus* | dom. caprine? |  | tooth left premolar | OP2; H2 ctx 21; phase 1 |
| KO181944 | Dali | *Ovis aries* | dom. caprine? |  | tooth right upper premolar | OP2; ctx 28 J5; phase 1 |
| KO181945 | Dali | *Ovis aries* | dom. caprine? |  | tooth left upper molar | OP2; ctx 20 J5; phase 1 |
| KO181947 | Dali | *Capra hircus* | dom. caprine? |  | tooth right upper molar | OP2; I1 ctx 21; phase 1 |
| KO181948 | Dali | *Ovis aries* | dom. caprine? |  | tooth right M/2 fragment | OP2; ctx 24 J1; phase 1 |
| KO181949 | Dali | *Ovis aries* | *Ovis aries* |  | tooth right M/2 | BR2; ctx 10 T033; phase 2+ |
| KO181960 | Begash | Fail | dom. caprine |  | bone; scapula | BG0014; ASIL 5211; IB Z102 ctx 14; phase 1a |
| KO181961 | Begash | *Ovis aries* | dom. caprine |  | bone; tibia | BG0002; ASIL 5219; IA y099 ctx 14; phase 1a |
| KO181962 | Begash | *Capra hircus* | dom. caprine |  | bone; humerus | BG0041; ASIL 5192; IA y100 ctx 15; phase 1a |
| KO181963 | Begash | *Ovis aries* | dom. caprine |  | bone; humerus | BG0029; ASIL 5186; IB y102 ctx 15; phase 1a |
| KO181964 | Begash | *Ovis aries* | dom. caprine |  | bone; radius | BG0013; ASIL 5209; IB z102 ctx 14; phase 1a |
| KO181965 | Begash | Fail | dom. caprine |  | bone; mandible | BG0005; ASIL 5222; IA z099 ctx 14; phase 1a |
| KO181966 | Begash | Fail | dom. caprine |  | bone; mandible | BG0037; ASIL 5205; IA z100 ctx 15; phase 1a |
| KO181967 | Begash | *Ovis aries* | *Ovis aries* |  | bone; ulna | BG0007; ASIL 5195; IB y102 ctx 14; phase 1a |
| KO181968 | Begash | *Ovis aries* | dom. caprine |  | bone; radius | BG0009; ASIL 5194; IB y101 ctx 14; phase 1a |
| KO181969 | Begash | *Ovis aries* | dom. caprine |  | bone; radius | BG0006; ASIL 5200; IA y100 ctx 14; phase 1a |
| KO181970 | Begash | *Ovis aries* | dom. caprine |  | bone; radius | BG0049; ASIL 5287; VC c116 ctx 14; phase 1b |
| KO181971 | Begash | Fail | dom. caprine |  | bone; mandible | BG0097; ASIL 5323; ID c103 ctx 8; phase 1b |
| KO181972 | Begash | Fail | dom. caprine |  | bone; scapula | BG0045; ASIL 5288; VC c120 ctx 16; phase 1b |
| KO181973 | Begash | Fail | dom. caprine |  | bone; tibia | BG0055; ASIL 5310; IB z102 ctx 6; phase 1b |
| KO181974 | Begash | *Ovis aries* | dom. caprine |  | bone; metatarsal | BG0090; ASIL 5305; ID c102 ctx 8; phase 1b |
| KO181975 | Begash | *Capra hircus* | dom. caprine |  | bone; tibia | BG0099; ASIL 5298; ID b101 ctx 10; phase 1b |
| KO181976 | Begash | *Capra sibirica* | dom. caprine | Y | bone; pubis | BG0117; ASIL 5320; VIA e120 ctx 16; phase 1b |
| KO181977 | Begash | *Ovis aries* | dom. caprine |  | bone; humerus | BG0053; ASIL 5266; IB z101 ctx6; phase 1b |
| KO181978 | Begash | *Ovis aries* | dom. caprine |  | bone; humerus | BG0107; ASIL 5267; IA y099 ctx 5; phase 1b |
| KO181979 | Dali | *Ovis aries* | dom. caprine? |  | bone; long bone | DL-OP2 I2 ctx 33; phase 1 |
| KO181980 | Dali | *Ovis aries* | dom. caprine? |  | bone; pubis | DL-OP2 J6 ctx 28; phase 1 |
| KO181981 | Dali | *Ovis aries* | dom. caprine? |  | bone; metapodial | DL-OP2 J6 ctx 28; phase 1 |
| KO181982 | Dali | *Ovis aries* | dom. caprine? |  | bone; metapodial | DL-OP2 J6 ctx 28; phase 1 |
| KO181983 | Dali | *Ovis aries* | dom. caprine? |  | bone; radius | DL-OP2 J6 ctx 28; phase 1 |
| KO181984 | Dali | *Ovis aries* | dom. caprine? |  | tooth left M2 fragment | DL-OP2 J0 ctx 24; phase 1 |
| KO181985 | Dali | *Ovis aries* | dom. caprine? |  | tooth P/3 fragment | DL-OP2 J0 ctx 24; phase 1 |
| KO181986 | Dali | *Ovis aries* | dom. caprine? |  | bone; metapodial | DL-OP2 J0 ctx 24; phase 1 |
| KO181988 | Dali | *Ovis aries* | dom. caprine? |  | bone; radius | ASIL: 4047; DL-OP2 I2 ctx 32; phase 1 |
| KO181989 | Dali | *Ovis aries* | dom. caprine? |  | bone; radius | ASIL: 4034; DL-OP2 J6 ctx 26; phase 1 |
| KO181996 | Dali | *Ovis aries* | dom. caprine? |  | bone; humerus | DL-OP2 J5 ctx 28; phase 1 |
| KO181997 | Dali | *Ovis aries* | dom. caprine? |  | bone; ulna | DL-OP2 J5 ctx 28; phase 1 |
| KO181998 | Dali | Fail | dom. caprine? |  | bone; humerus | DL-OP2 J5 ctx 28; phase 1 |
| KO181999 | Dali | *Ovis aries* | dom. caprine? |  | bone; metatarsal | DL-OP2 J5 ctx 28; phase 1 |
| KO182316 | Begash | *Ovis aries* | dom. caprine |  | bone; radius | BG0031; ASIL: 52010; IB; y102; ctx 15; phase 1a |
| KO182317 | Begash | *Ovis aries* | dom. caprine |  | bone; humerus | BG0040; ASIL: 5183; IB; y101; ctx 19; phase 1a |
| KO182318 | Begash | Fail | dom. caprine |  | bone; rib | BG0021; ASIL: 5208; IC; a099; ctx 15; phase 1a |
| KO182319 | Begash | *Ovis aries* | dom. caprine |  | bone; humerus | BG0033; ASIL: 5197; IB; z101; ctx15; phase 1a |
| KO182320 | Begash | Fail | dom. caprine |  | bone; rib | BG0041a; ASIL: 5218; IB; z102; ctx 23; phase 1a |
| KO182321 | Begash | *Ovis aries* | *Ovis aries* |  | bone; metacarpal | BG0025; ASIL: 5201; IC; a100; ctx 14; phase 1a |
| KO182322 | Begash | *Ovis aries* | dom. caprine |  | bone; metatarsal | BG0027; ASIL: 5198; IC; b100; ctx 14; phase 1a |
| KO182323 | Begash | *Ovis aries* | dom. caprine |  | bone; metacarpal | BG0019; ASIL: 5212; IC; b100; ctx 15; phase 1a |
| KO182324 | Begash | *Ovis aries* | dom. caprine |  | bone; metacarpal | BG0008; ASIL 5203; IB; y102; ctx 14; phase 1a |
| KO182325 | Begash | *Ovis aries* | dom. caprine |  | bone; radius | BG0012; ASIL: 5193; IB; z102; ctx 14; phase 1a |
| KO182326 | Begash | *Ovis aries* | *Ovis aries* |  | bone; scapula | BG0010; ASIL: 5202; IB; z102; ctx 14; phase 1a |
| KO182327 | Begash | *Capra hircus* | dom. caprine |  | bone; metatarsal | BG0017; ASIL: 5221; IC; b099; ctx 15; phase 1a |

**Table S3.** Summary of herbivore teeth sampled for δ^13^C_apa_ and δ^18^O_apa_ values. Raw data are provided in dataset S3.

| Tooth ID | Site | Period | Phase | Taxon | Element | Symmetry | # samples | ^14^C sample |
| --- | --- | --- | --- | --- | --- | --- | --- | --- |
| T132 | Begash | Early Bronze Age | 1a | Caprine | M/2 | right | 11 | Poz-88835 |
| T131 | Begash | Early Bronze Age | 1a | Caprine | M/3 | right | 8 | Poz-88835 |
| T031 | Begash | Early Bronze Age | 1a | Caprine | M/3 | left | 19 |  |
| T001 | Begash | Middle Bronze Age | 1b | Caprine | M/3 |  | 10 |  |
| T003 | Begash | Middle Bronze Age | 1b | Caprine | M/3 | left | 18 |  |
| T008 | Begash | Middle Bronze Age | 1b | Caprine | M/3 | left | 6 |  |
| T032 | Begash | Late Bronze Age | 2 | Caprine | M/2 | left | 13 |  |
| T112 | Begash | Late Bronze Age | 2 | Caprine | M/3 | right | 16 |  |
| T113 | Begash | Late Bronze Age | 2 | Caprine | M/2 | left | 10 |  |
| T114 | Begash | Late Bronze Age | 2 | Caprine | M/2 | right | 14 |  |
| T115 | Begash | Late Bronze Age | 2 | Caprine | M/2 | left | 8 |  |
| T014 | Begash | Iron Age | 3b | Caprine | M/3 |  | 14 |  |
| T016 | Begash | Iron Age | 3b | Caprine | M/3 | right | 18 |  |
| T018 | Begash | Iron Age | 3b | Caprine | M/3 | left | 14 |  |
| T019 | Begash | Iron Age | 3b | Caprine | M/3 | right | 15 |  |
| T030 | Begash | Iron Age | 3b | Caprine | M/3 | right | 15 |  |
| T146 | Begash | Iron Age | 3b | Caprine | M/3 | right | 15 |  |
| T147 | Begash | Iron Age | 3b | Caprine | M/2 | left | 17 |  |
| T022 | Dali | Early Bronze Age | 1 | Caprine | M/3 | left | 20 |  |
| T027 | Dali | Early Bronze Age | 1 | Caprine | M/3 | left | 14 | Beta-484122 |
| T050 | Dali | Early Bronze Age | 1 | Caprine | M/3 |  | 5 |  |
| T085 | Dali | Early Bronze Age | 1 | Caprine | M2 | right | 8 |  |
| T026 | Dali | Late Bronze Age | 2 | Caprine | M/3 | left | 9 |  |
| T023 | Dali | Late Bronze Age | 2 | Caprine | M/3 | right | 18 |  |
| T029 | Dali | Late Bronze Age | 2 | Caprine | M/3 | right | 20 |  |
| T033 | Dali | Final Bronze Age | 3 | Caprine | M/2 | right | 24 |  |
| T025 | Tasbas | Late Bronze Age | 2b | Caprine | M/3 | right | 5 |  |
| T024 | Tasbas | Late Bronze Age | 2a | Caprine | M/3 | right | 24 |  |
| T102 | Begash | Middle Bronze Age | 1b | Bos | M/2 | right | 14 |  |
| T103 | Begash | Middle Bronze Age | 1b | Bos | M/2 | right | 12 |  |
| T105 | Begash | Middle Bronze Age | 1b | Bos | M/1 | right | 10 |  |
| T110 | Begash | Late Bronze Age | 2 | Bos | M/2 | right | 9 |  |
| T111 | Begash | Late Bronze Age | 2 | Bos | M/3 | right | 10 |  |
| T117 | Begash | Late Bronze Age | 2 | Bos | M/3 | right | 10 |  |
| T138 | Begash | Late Bronze Age | 2 | Bos | M/2 | left | 10 |  |
| T013 | Begash | Iron Age | 3b | Bos | M/2 |  | 8 |  |
| T140 | Begash | Iron Age | 3b | Bos | M/2 | right | 10 |  |
| T071 | Dali | Early Bronze | 1 | Bos | M/3 | left | 16 |  |
| T072 | Dali | Early Bronze | 1 | Bos | M/3 | right | 16 |  |
| T096 | Dali | Early Bronze | 1 | Bos | M/2 | right | 6 |  |
| T073 | Dali | Middle Bronze Age | 2 | Bos | M/2 | right | 15 |  |
| T077 | Dali | Middle Bronze Age | 2 | Bos | M/2 |  | 10 |  |
| T101 | Tasbas | Final Bronze Age | 3 | Bos | M/2 | left | 11 |  |
| T184 | Begash | Middle Bronze Age | 1b | Cervus | M/3 | left | 5 |  |
| T127 | Begash | Iron Age | 3b | Cervus | M/3 | right | 17 |  |
| T129 | Begash | Historic | 6 | Cervus | M/2 | right | 14 |  |
| T130 | Begash | Historic | 6 | Cervus | M/3 | right | 7 |  |
| T157 | Begash | Historic | 6 | Caprine | M/3 | right | 13 |  |
| T020 | Bayan-Zherek | Modern |  | Caprine | M/2 | left | 21 |  |
| T021 | Bayan-Zherek | Modern |  | Caprine | M/3 | left | 17 |  |
| T191 | Bozshakol | Late Bronze Age | 1 | Saiga | M/2 | left | 2 |  |
| T192 | Bozshakol | Late Bronze Age | 1 | Saiga | M/3 | left | 4 |  |

**Table S4.** Summary of bone collagen samples for δ^13^C and δ^15^N and number of samples with acceptable C:N ratios and percent elemental composition (Main text: Materials and Methods). Poor collagen preservation is observed at Begash phases 3a and 3b. Raw data are provided in dataset S4.

|  | Site | Dali | | Dali/Tasbas | | Begash | | | | | | |  |  |
| --- | --- | --- | --- | --- | --- | --- | --- | --- | --- | --- | --- | --- | --- | --- |
|  | Phase | 1 | | 2 | | 1a | | 1b | | 2 | | 3a/b | | |
|  |  | Total | Accepted | Total | Accepted | Total | Accepted | Total | Accepted | Total | Accepted | Total | Accepted | |
| Domestic | Caprine | 4 | 4 | 9 | 9 | 23 | 23 | 33 | 33 | 35 | 30 | 44 | 13 | |
|  | Cattle | 6 | 6 | 1 | 1 | 11 | 11 | 19 | 19 | 20 | 18 | 23 | 6 | |
| Wild | Red deer |  |  | 1 | 0 | 2 | 2 | 8 | 8 | 3 | 3 | 13 | 6 | |
|  | Ibex |  |  | 1 | 1 |  |  | 1 | 1 |  |  |  |  | |
|  | Argali |  |  |  |  |  |  |  |  | 1 | 1 | 1 | 0 | |
|  | Human | 1 | 1 | 1 | 1 |  |  |  |  | 1 | 1 | 1 | 1 | |


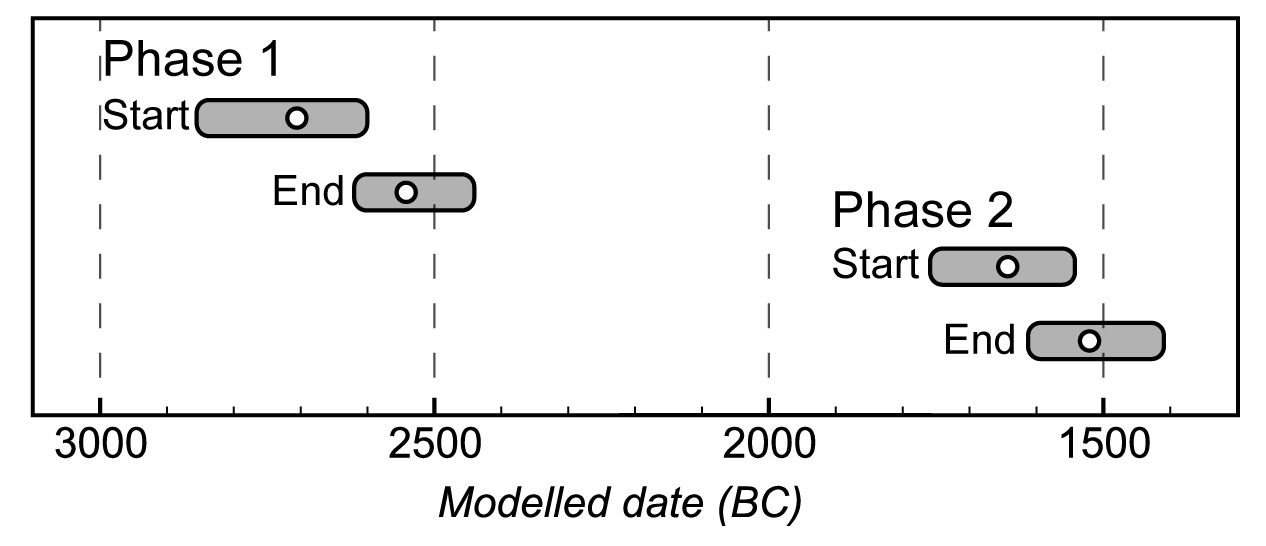


**Fig. S1.** Radiocarbon chronology of early and middle Bronze Age occupations of the Dali settlement. Start and end dates for each phase are shown as 95% posterior probability ranges with means as white circles in calibrated years BC. Phase 1 dates were measured on charcoal (n=4), animal bone/teeth (n=2), and human bone (n=1). Phase 2 dates were measured on charcoal (n=2) and animal bone (n=3). Table S1 contains underlying radiocarbon dates for Dali, Tasbas, and Begash.


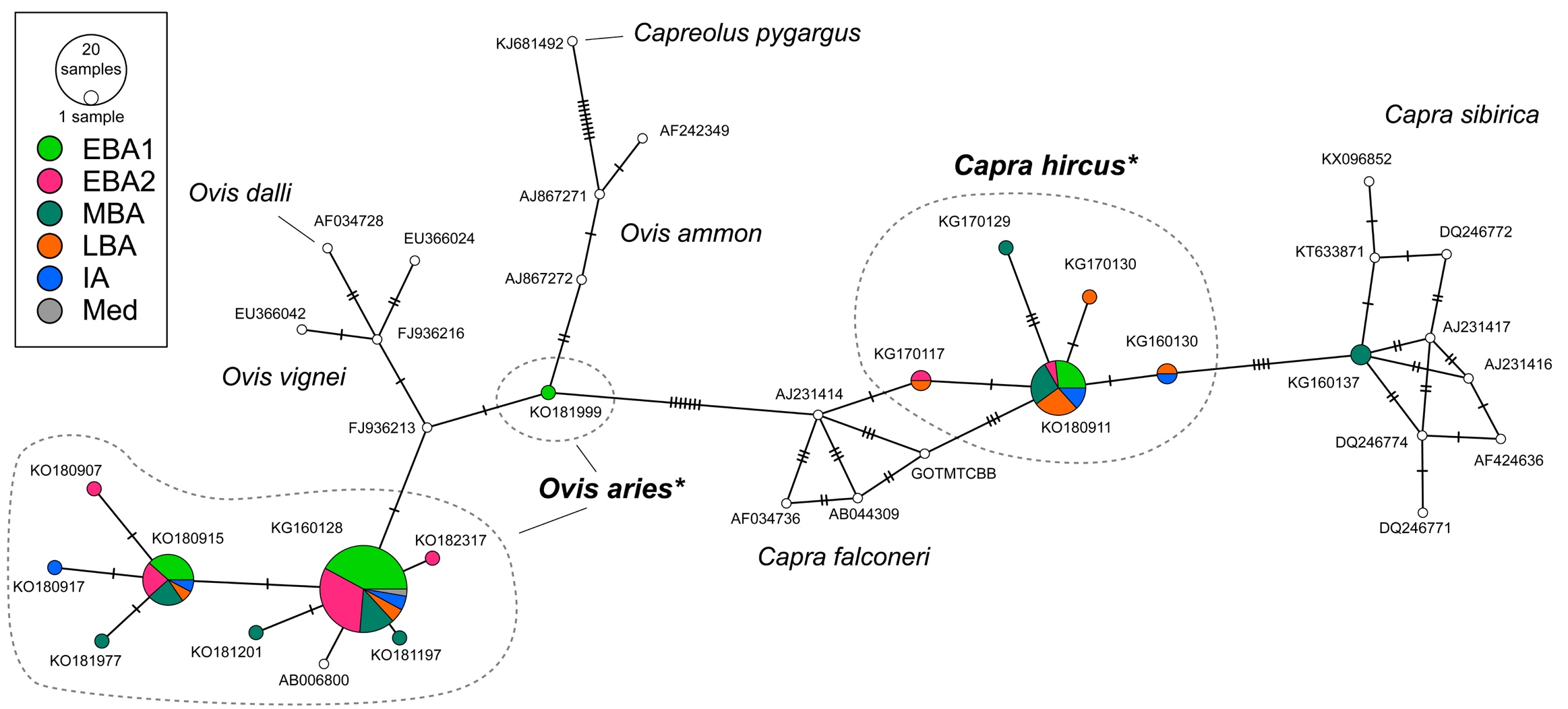


**Fig. S2.** Minimum spanning network of sample *MT-CYB* sequences (colourful nodes indicating relative abundance by period) and reference sequences of *Capra*/*Ovis* spp. and distant *Capreolus* sp. (white nodes). Number of mutations are indicated by hash marks along edges. Domesticated sheep and goat (*Ovis aries** and *Capra hircus**) haplotypes dominate the sample set from all cultural periods, notably early Bronze Age layers of Dali (EBA1) and Begash (EBA2). Two samples yielded *Capra sibirica* haplotypes from middle Bronze Age (MBA) layers. See table S2 and dataset S1.

**
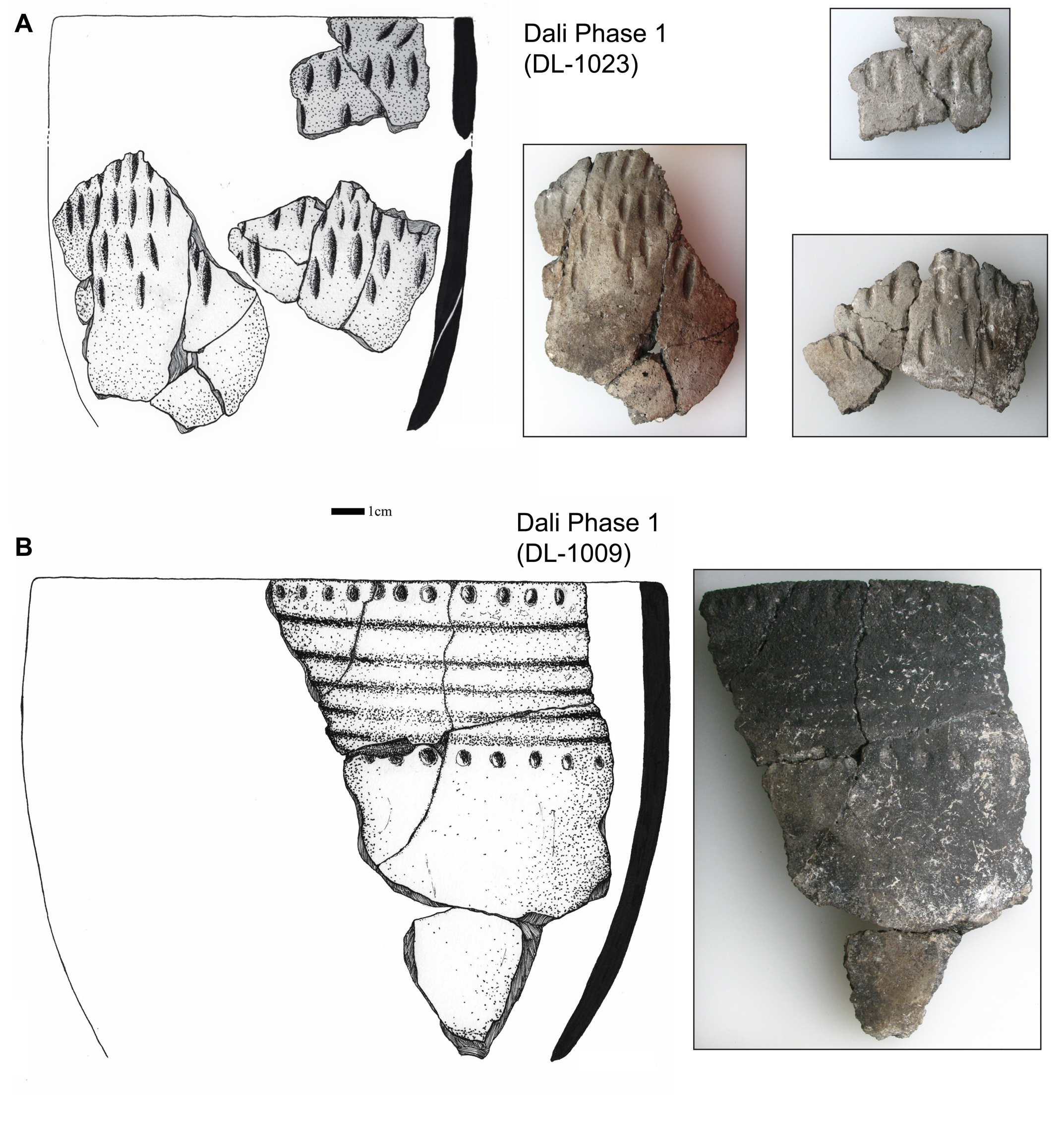
**

**Fig. S3.** Examples of pottery from Dali Phase 1, showing similarity to third millennium BC cultures from the Altai region [17]. Styles consist of individual impressions of (A) almond or (B) round shapes and shallow depressions made in bands around the body of pots.

**
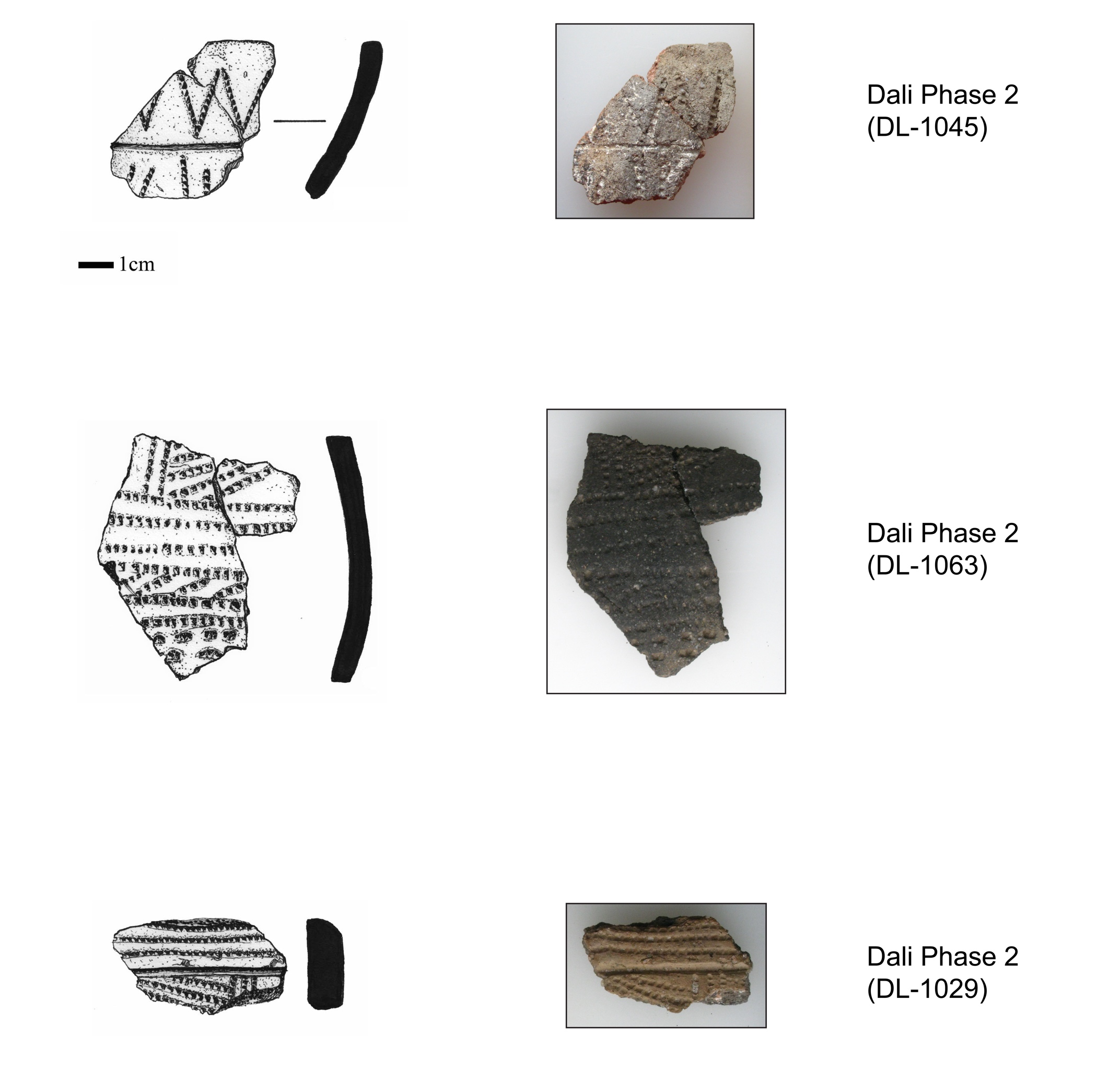
**

**Fig. S4.** Examples of pottery of Dali Phase 2, demonstrating diverse styles of comb-incised steppe ceramics that are ubiquitous throughout the Eurasian steppe region in the mid-late second millennium BC. Comb impressions that are oriented in repeating geometric patterns, typically bands and triangles, are one of the most common features of varied cultural groups associated with the “Andronovo” archaeological horizon [18].

**
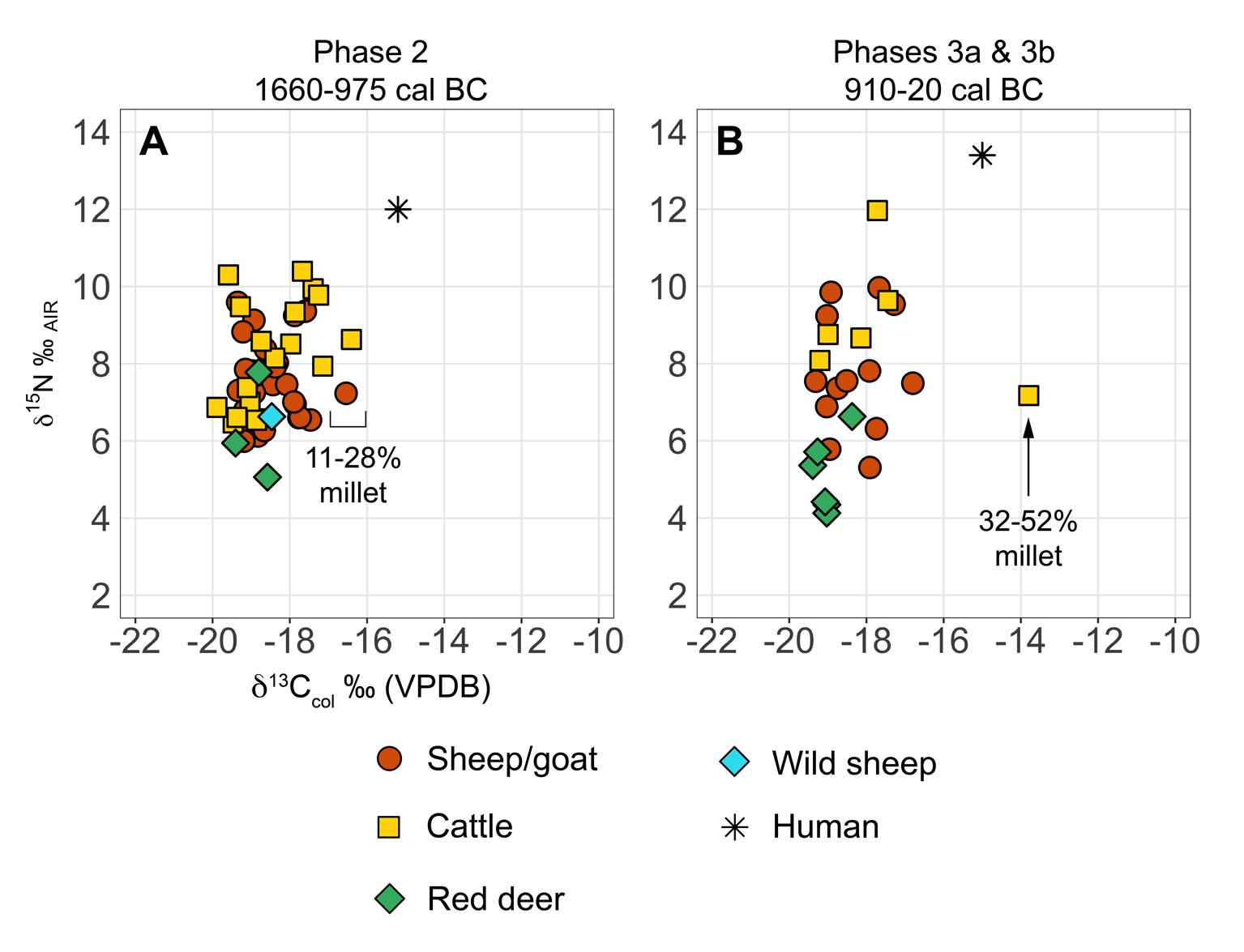
**

**Fig. S5.** δ^13^C_col_ and δ^15^N_col_ values of livestock, wild herbivores, and humans from (A) late Bronze Age and (B) Iron Age Begash, including MixSAIR estimates of percent C_4_ dietary intake. Raw data are in dataset S4.

**
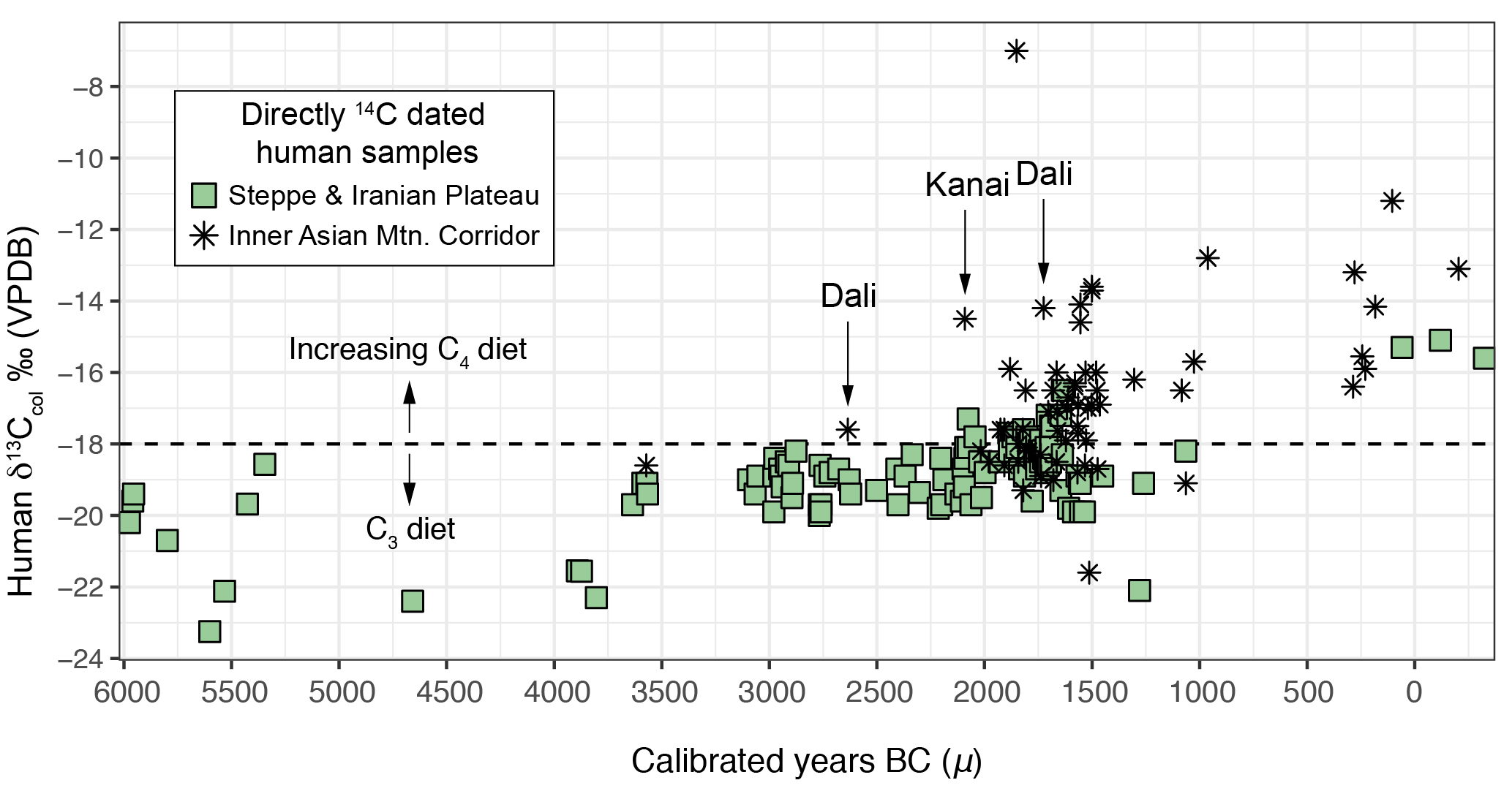
**

**Fig. S6.** Plot of δ^13^C values against calibrated ^14^C determinations from human samples in the IAMC and western and steppe regions [12,19–22] (n=174; dataset S5). The intensification of millet consumption is detected isotopically by ~2100 cal BC, but preceding millennia are poorly characterized. Other isotopic studies, most relying on relative chronologies based on material culture, suggest a widespread adoption of millet as a dietary staple by steppe societies by the Iron Age, which extends into the medieval period [23–27]. Site locations proving carbon isotopic data are mapped in figure S7.


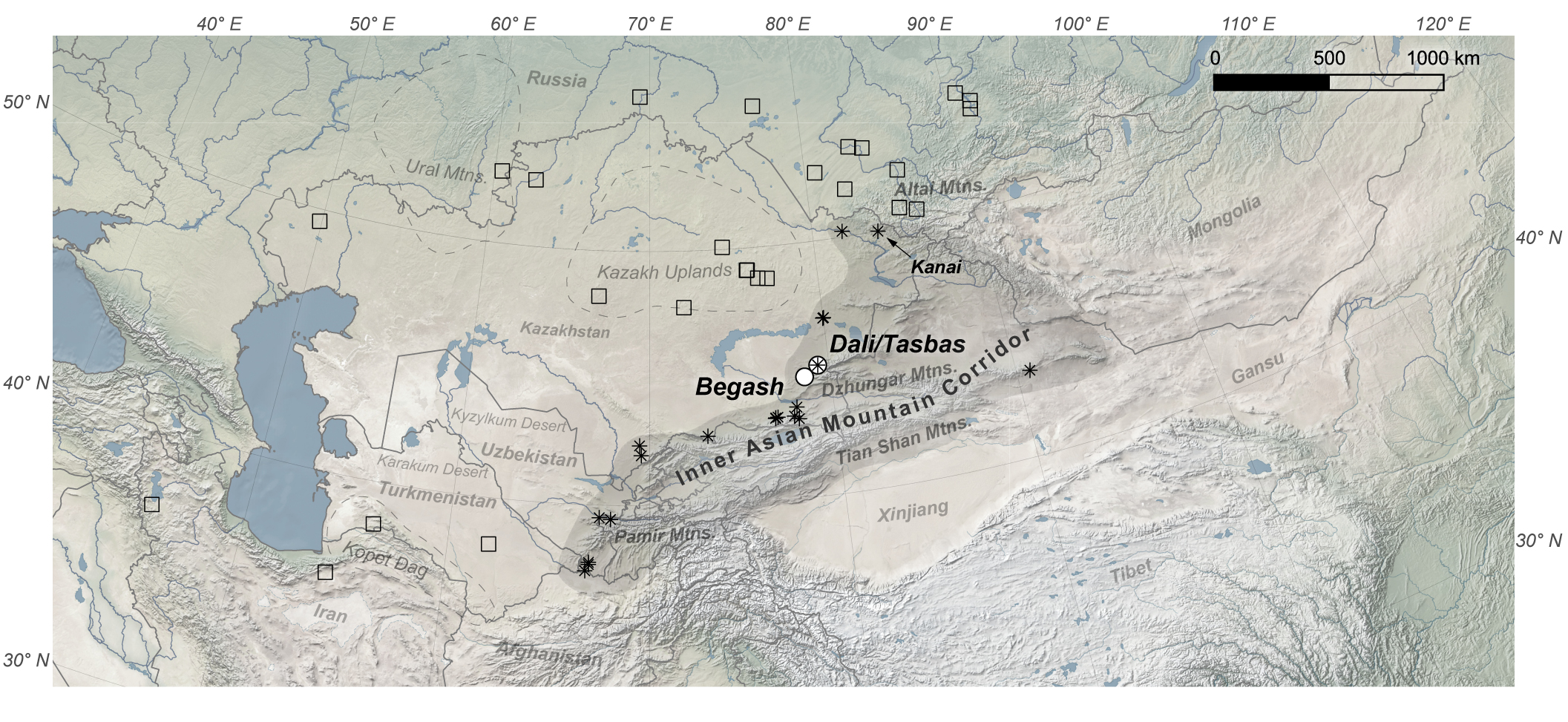


**Fig. S7.** Locations of sites providing human carbon isotopic data in Fig. S5. Boxes denote sites in the steppe and Iranian Plateau, and asterisks denote sites in the Inner Asian Mountain Corridor. Data are in dataset S5.

**Dataset S1.** Sample and reference mitochondrial cytochrome *b* sequences (110-bp) in FASTA format (DatasetS1_MT-CYB_sequences_final.fasta).

**Dataset S2.** δ^13^C values of broomcorn millet leaves and reference taxa displayed in Fig. 3, including corrections used to account for Suess effect for modern samples and trophic level enrichment between dietary sources and tissues (DatasetS2_d13c_values_vegetation_reconstruction_final.csv).

**Dataset S3.** δ^13^C_apa_ and δ^18^O_apa_ values and incremental distances of tooth enamel from domesticated and wild herbivores (DatasetS3_faunal_tooth_enamel_C_O_isotope_data_final.csv).

**Dataset S4.** δ^13^C_col_ and δ^15^N_col_ values from samples of bone collagen from domesticated and wild herbivores and humans from Dali, Tasbas, and Begash, including modern steppe tortoise (DatasetS4_KILT_C_N_isotope_data_final.csv).

**Dataset S5.** δ^13^C_col_ and δ^15^N_col_ values and radiocarbon dates of humans from the IAMC and wider steppe region (DatasetS5_human_14C_d13C_final.csv).

**References**

1. Leigh JW, Bryant D. 2015 popart: full-feature software for haplotype network construction. *Methods Ecol. Evol.* **6**, 1110–1116. (doi:10.1111/2041-210X.12410)

2. An C-B, Dong W, Li H, Zhang P, Zhao Y, Zhao X, Yu S-Y. 2015 Variability of the stable carbon isotope ratio in modern and archaeological millets: evidence from northern China. *J. Archaeol. Sci.* **53**, 316–322. (doi:10.1016/j.jas.2014.11.001)

3. Suess HE. 1955 Radiocarbon Concentration in Modern Wood. *Science* **122**, 415–417. (doi:10.1126/science.122.3166.415-a)

4. Cerling TE, Harris JM. 1999 Carbon isotope fractionation between diet and bioapatite in ungulate mammals and implications for ecological and paleoecological studies. *Oecologia* **120**, 347–363.

5. Passey BH, Robinson TF, Ayliffe LK, Cerling TE, Sponheimer M, Dearing MD, Roeder BL, Ehleringer JR. 2005 Carbon isotope fractionation between diet, breath CO_2_, and bioapatite in different mammals. *J. Archaeol. Sci.* **32**, 1459–1470. (doi:10.1016/j.jas.2005.03.015)

6. Jürgensen J, Drucker DG, Stuart AJ, Schneider M, Buuveibaatar B, Bocherens H. 2017 Diet and habitat of the saiga antelope during the late Quaternary using stable carbon and nitrogen isotope ratios. *Quat. Sci. Rev.* **160**, 150–161. (doi:10.1016/j.quascirev.2017.01.022)

7. Zazzo A, Balasse M, Passey BH, Moloney AP, Monahan FJ, Schmidt O. 2010 The isotope record of short- and long-term dietary changes in sheep tooth enamel: Implications for quantitative reconstruction of paleodiets. *Geochim. Cosmochim. Acta* **74**, 3571–3586. (doi:10.1016/j.gca.2010.03.017)

8. Hedges REM. 2003 On bone collagen—apatite-carbonate isotopic relationships. *Int. J. Osteoarchaeol.* **13**, 66–79. (doi:10.1002/oa.660)

9. Lee-Thorp JA, Sealy JC, van der Merwe NJ. 1989 Stable carbon isotope ratio differences between bone collagen and bone apatite, and their relationship to diet. *J. Archaeol. Sci.* **16**, 585–599. (doi:10.1016/0305-4403(89)90024-1)

10. Froehle AW, Kellner CM, Schoeninger MJ. 2010 FOCUS: effect of diet and protein source on carbon stable isotope ratios in collagen: follow up to Warinner and Tuross (2009). *J. Archaeol. Sci.* **37**, 2662–2670. (doi:10.1016/j.jas.2010.06.003)

11. Steinitz R, Lemm JM, Pasachnik SA, Kurle CM. 2016 Diet-tissue stable isotope (*Δ*^13^C and *Δ*^15^N) discrimination factors for multiple tissues from terrestrial reptiles: Stable C and N isotope discrimination factors for reptiles. *Rapid Commun. Mass Spectrom.* **30**, 9–21. (doi:10.1002/rcm.7410)

12. Narasimhan VM *et al.* 2018 The Genomic Formation of South and Central Asia. *bioRxiv* , 292581. (doi:10.1101/292581)

13. Spengler RN, Frachetti MD, Doumani PN. 2014 Late Bronze Age agriculture at Tasbas in the Dzhungar Mountains of eastern Kazakhstan. *Quat. Int.* **348**, 147–157. (doi:10.1016/j.quaint.2014.03.039)

14. Doumani PN, Frachetti MD, Beardmore R, Schmaus TM, Spengler RN, Mar’yashev AN. 2015 Burial ritual, agriculture, and craft production among Bronze Age pastoralists at Tasbas (Kazakhstan). *Archaeol. Res. Asia* **1–2**, 17–32. (doi:10.1016/j.ara.2015.01.001)

15. Frachetti MD, Mar’yashev AN. 2007 Long-Term Occupation and Seasonal Settlement of Eastern Eurasian Pastoralists at Begash, Kazakhstan. *J. Field Archaeol.* **32**, 221–242.

16. Spengler RN, Frachetti M, Doumani P, Rouse L, Cerasetti B, Bullion E, Mar’yashev A. 2014 Early agriculture and crop transmission among Bronze Age mobile pastoralists of Central Eurasia. *Proc. R. Soc. B Biol. Sci.* **281**, 20133382. (doi:10.1098/rspb.2013.3382)

17. Doumani P. 2014 Ceramic Technology of Bronze Age Nomadic Pastoralists of Semirech’ye, Kazakhstan. Doctoral Dissertation, Washington University in St. Louis, St. Louis.

18. Frachetti MD. 2008 *Pastoralist landscapes and social interaction in bronze age Eurasia*. Berkeley: University of California Press.

19. Motuzaite Matuzeviciute G, Lightfoot E, O’Connell TC, Voyakin D, Liu X, Loman V, Svyatko S, Usmanova E, Jones MK. 2015 The extent of cereal cultivation among the Bronze Age to Turkic period societies of Kazakhstan determined using stable isotope analysis of bone collagen. *J. Archaeol. Sci.* **59**, 23–34. (doi:10.1016/j.jas.2015.03.029)

20. Motuzaite-Matuzeviciute G, Kiryushin YF, Rakhimzhanova SZ, Svyatko S, Tishkin AA, O’Connell TC. 2016 Climatic or dietary change? Stable isotope analysis of Neolithic–Bronze Age populations from the Upper Ob and Tobol River basins. *The Holocene* , 0959683616646843. (doi:10.1177/0959683616646843)

21. Svyatko SV, Polyakov AV, Soenov VI, Stepanova NF, Reimer PJ, Ogle N, Tyurina EA, Grushin SP, Rykun MP. 2017 Stable isotope palaeodietary analysis of the Early Bronze Age Afanasyevo Culture in the Altai Mountains, Southern Siberia. *J. Archaeol. Sci. Rep.* **14**, 65–75. (doi:10.1016/j.jasrep.2017.05.023)

22. Wang T, Wei D, Chang X, Yu Z, Zhang X, Wang C, Hu Y, Fuller BT. 2017 Tianshanbeilu and the Isotopic Millet Road: Reviewing the late Neolithic/Bronze Age radiation of human millet consumption from north China to Europe. *Natl. Sci. Rev.*

23. Ananyevskaya E *et al.* 2017 Early indicators to C4 plant consumption in central Kazakhstan during the Final Bronze Age and Early Iron Age based on stable isotope analysis of human and animal bone collagen. *Archaeol. Res. Asia* (doi:10.1016/j.ara.2017.12.002)

24. Lightfoot E, Motuzaite-Matuzeviciute G, O’Connell TC, Kukushkin IA, Loman V, Varfolomeev V, Liu X, Jones MK. 2015 How ‘Pastoral’ is Pastoralism? Dietary Diversity in Bronze Age Communities in the Central Kazakhstan Steppes. *Archaeometry* **57**, 232–249. (doi:10.1111/arcm.12123)

25. Svyatko SV, Schulting RJ, Mallory J, Murphy EM, Reimer PJ, Khartanovich VI, Chistov YK, Sablin MV. 2013 Stable isotope dietary analysis of prehistoric populations from the Minusinsk Basin, Southern Siberia, Russia: a new chronological framework for the introduction of millet to the eastern Eurasian steppe. *J. Archaeol. Sci.* **40**, 3936–3945. (doi:10.1016/j.jas.2013.05.005)

26. Lightfoot E, Liu X, Jones MK. 2013 Why move starchy cereals? A review of the isotopic evidence for prehistoric millet consumption across Eurasia. *World Archaeol.* **45**, 574–623. (doi:10.1080/00438243.2013.852070)

27. Hermes TR, Frachetti MD, Bullion EA, Maksudov F, Mustafokulov S, Makarewicz CA. 2018 Urban and nomadic isotopic niches reveal dietary connectivities along Central Asia’s Silk Roads. *Sci. Rep.* **8**, 5177. (doi:10.1038/s41598-018-22995-2)
